# Satisfaction with dental care among patients who receive invasive or non-invasive treatment for non-cavitated early dental caries: findings from one region of the National Dental PBRN

**DOI:** 10.1186/s12903-017-0363-8

**Published:** 2017-03-27

**Authors:** Sonya T. Mitchell, Ellen Funkhouser, Valeria V. Gordan, Joseph L. Riley, Sonia K. Makhija, Mark S. Litaker, Gregg H. Gilbert, National Dental PBRN Collaborative Group

**Affiliations:** 1 University of Alabama at Birmingham, SDB Room 609; Box 48, 1720 Second Avenue South, Birmingham, AL 35294-0007 USA; 2 Department of Preventive Medicine, School of Medicine, University of Alabama at Birmingham, Birmingham, AL USA; 3 Department of Restorative Dental Sciences, College of Dentistry, University of Florida, Gainesville, FL USA

**Keywords:** Patient satisfaction, Practice-based research, Dentistry, Non-invasive treatment, Invasive treatment

## Abstract

**Background:**

The objectives were to: (1) quantify patient satisfaction with treatment for early dental caries overall, and according to whether or not (2a) the patient received invasive treatment; (2b) was high-risk for dental caries, and had dental insurance; and (3) encourage practitioners to begin using non-invasive approaches to early caries management.

**Methods:**

Ten practitioners recorded patient, lesion, and treatment information about non-cavitated early caries lesions. Information on 276 consecutive patients with complete data was included, who received either non-invasive (no dental restoration) or invasive (dental restoration) treatment. Patients completed a patient satisfaction questionnaire and were classified as dissatisfied if they did not “agree” or “strongly agree” with any of 14 satisfaction items.

**Results:**

Patients had a mean (± SD) age of 41.8 (±15.8) years, 64% were female and 88% were white. Twenty-five percent (*n* = 68) were dissatisfied in at least one of the 14 satisfaction items. Satisfaction levels did not significantly vary by patient’s gender, race, caries risk category, or affected tooth surface location. Overall, 11% (28 of 276) received invasive treatment; satisfaction did not differ between patients who had invasive or non-invasive treatment. Seven patients received invasive treatment at their request even though that was not what their practitioner recommended; 5 out of 6 were satisfied with their treatment nonetheless.

**Conclusions:**

About one-fourth of patients treated for non-cavitated early caries were dissatisfied with at least some aspect of their dental care experience. Satisfaction of patients who received invasive treatment did not differ from those who received non-invasive treatment.

## Background

Patient satisfaction can impact a patient’s likelihood of choosing a dentist, arranging and keeping appointments, and complying with dentists’ instructions [1–8]. Therefore, patient satisfaction is a salient component of health care and one that has the potential to impact treatment outcomes. Literature supports the notion that a healthy dentist-patient relationship contributes to higher patient satisfaction, and a recent review concluded that patients were more positive about a dental practice when they felt that the dental team listened to their concerns [9]. Research has shown that patient education and decision aids can improve the provider-patient relationship and improve decision-related outcomes [9, 10]. Therefore, the current study developed patient education materials specific to early caries management, provided them to participating dentists, and advised them regarding their use with patients.

Levels of satisfaction may differ by dental procedure and by which clinical populations are being served. Unfortunately, most studies have focused on dental visits in general [11, 12] and have sampled from the community without regard to time lag or the type of dental care received [3, 13–15]. Additionally, many studies have taken place in university dental clinics, a setting for which generalizability to other practice settings may be debatable [2, 16–18]. Consequently, with the exception of prosthetic [19, 20] and orthodontic [21] procedures, there is little information about dental patient satisfaction with specific types of dental procedures. The placement of dental restorations is one of the most commonly performed procedures by general dentists [22, 23], yet little is known about patient satisfaction with visits having to do with caries management and dental restorations [5, 12, 24].

There is substantial variation in caries diagnosis and management [25, 26], with many dentists choosing an invasive approach (i.e., a dental restoration) rather than non-invasive treatment methods (e.g., “watchful monitoring” combined with prevention) for early caries. More recent published literature supports the benefits of minimally invasive dentistry. For example, an FDI World Dental Federation policy statement on managing dental caries supports minimally invasive operative interventions [27, 28]. Also, the American Academy of Pediatric Dentistry’s published Guideline on Restorative Dentistry recommends active surveillance of non-cavitated carious lesions with preventive management [29]. Nonetheless, third party payers reimburse surgical procedures at a higher rate than the surveillance procedures that are consistent with evidence-based dentistry policy statements. The decision to place the first restoration on a tooth surface is an important one because it often is the beginning of an unfortunate cycle of restoration replacement over subsequent decades in which each succeeding restoration is progressively larger, ultimately leading to a large restoration [30–32] that places the tooth at increased risk for extraction [33, 34]. Approaches that delay placement of the first restoration may be a key source of improving the long-term effectiveness of dental care and therefore its quality [33, 35].

The National Dental Practice-Based Research Network (National Dental PBRN) is a consortium of dental practices and dental organizations focused on improving the scientific basis for clinical decision-making [36, 37]. This project initiated a line of research that was ultimately aimed at improving the quality of care in dental practices by introducing evidence-based decision-making information into routine practice. This project aimed to capitalize on information collected in two previous network studies of early caries diagnosis and treatment [25, 26, 38] and generate basic information for future studies that would be designed to decrease variation in caries treatment. Beginning as a small-scale study limited to one network region, 10 Alabama practitioners were identified who would intervene invasively (i.e., do a dental restoration) on a non-cavitated caries lesion that was only into the enamel. These practitioners received in-office education about non-invasive treatment options and the latest scientific evidence about them.

For the purposes of the current study, early caries is defined as non-cavitated lesions confined to the enamel. Furthermore, non-invasive treatment is defined as watchful monitoring combined with prevention: oral hygiene instructions, diet counseling, fluoride treatments (rinse, gel, or paste), antibacterial rinses, varnishes or sealants. Air abrasion prior to sealing an early carious lesion was considered non-invasive as well.

The objectives for this study were to: (1) quantify patient satisfaction with treatment for early dental caries overall, and according to whether or not (2a) the patient received invasive treatment; (2b) was high-risk for dental caries, and had dental insurance; and (3) encourage practitioners to begin using non-invasive approaches to early caries management.

## Methods

### Multi-phased approach

**Fig. 1:**
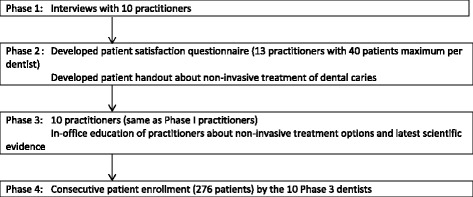
Flow chart describing the multi-phased approach to this study

The long-range goal of this line of research was to improve the quality of dental care by fostering movement of the latest scientific advances into routine clinical practice, specifically in the area of treatment of non-cavitated early caries. This plan involved four phases as illustrated in Fig. 1. Beginning as a small-scale study limited to one network region, the plan commenced after 10 Alabama practitioners were identified who had reported during a questionnaire study about caries diagnosis and caries treatment [26, 38] that they would intervene invasively (i.e., do a dental restoration) on a non-cavitated caries lesion that was only into the enamel. In a subsequent clinical study of dental restorations done on previously-unrestored tooth surfaces [25], we confirmed that these same 10 practitioners did indeed restore some non-cavitated lesions that were only into the enamel.

Phase 1 involved semi-structured interviews with these 10 practitioners. During the interviews, these practitioners expressed several barriers to providing non-invasive treatment. These were: (1) patients need help understanding why non-invasive treatment is advantageous; (2) some practitioners fear that if non-invasive treatment leads to an outcome that patients perceive as very negative (e.g., advanced caries), then the patient (or another local dentist from whom the patient decides to begin receiving treatment) will harbor negative attitudes toward the practitioner; (3) practitioners did not want to be seen as outliers, and therefore would like to be able to provide a brochure to help legitimize and validate the non-invasive treatment approach. These Phase 1 results informed the activities that occurred in Phase 2.

Phase 2 entailed developing a patient satisfaction questionnaire specific to early caries management; developing a patient handout about non-invasive treatment of dental caries; and obtaining input from dentists and patients about how best to use the handout to educate patients about non-invasive treatment. Phase 2 obtained input from these 10 practitioners and 30 of their patients during the development of the handout and its feasibility testing, as well as from 10 patients and 3 dentists in the University of Alabama at Birmingham faculty practice.

Phase 3 involved in-office education of the practitioners about non-invasive treatment options and the latest scientific evidence about them. The educational materials used in Phase 3 are publicly available at the network’s web site [39]. One objective of this phase was to encourage these 10 practitioners to begin using non-invasive approaches to early caries management.

Phase 4 involved demonstrating to dentists – by engaging consecutive patients who have non-cavitated dental caries from their own practices during actual clinical encounters – that patient satisfaction was comparable or higher among patients who received non-invasive treatment as compared to invasive treatment. The data collection forms (Consecutive Patient Log; Caries Risk Assessment Form; Patient and Clinical Characteristics Data Collection Form; Patient Satisfaction Survey) used during this phase are publicly available at the network’s web site [39].

### Phase 4 study procedures

Practitioners explained the study to eligible consecutive patients during the data collection period. To be eligible, a patient had to have at least one non-cavitated early caries lesion, defined as confined to the enamel based on the practitioner’s assessment of the clinical and/or radiographic appearance. Additionally, the patient had to be 19 years old or older and had to receive treatment for this lesion (either invasive or non-invasive treatment) during the current appointment or at the next appointment. If the patient provided informed consent, he or she was enrolled in the study, given the patient education brochure, and asked to read the brochure. The practitioner then explained or reviewed the content of the brochure with the patient. The practitioner next completed a Caries Risk Assessment Form about the patient’s caries status.

The practitioner next recorded information about patient characteristics (e.g., the patient’s gender, age, race, presence or absence of dental insurance, caries risk, and number of early caries lesions). Practitioners also recorded tooth-level characteristics (which surfaces had non-cavitated enamel caries; depth of the lesion), as well as what treatment was selected (monitoring; oral hygiene instruction; applying/prescribing fluoride; applying varnish; sealant placement; enameloplasty; preventive resin restoration; full restoration; other). “Preventive resin restoration” and “full restoration” were classified as invasive treatment; all other options were classified as non-invasive treatment. Patients were informed about monitoring lesions and instructions were documented in the patient’s treatment record. Patients received an explanation of the advantages and prognosis of caries preventive programs. If the practitioner recommended a non-invasive treatment to the patient, but the patient requested an invasive treatment anyway, the practitioner recorded the reason (does not have time for home care; does not believe that non-invasive treatment works; prefers a quick-fix to the problem; other). After the patient treatment was completed, the patient was given a Patient Satisfaction Survey Questionnaire (Appendix) and asked to mail that to the project’s research assistant at the University of Alabama at Birmingham. All treatment options offered to the patient were considered standard of care; yet, the patients selected their preferred treatment after receiving an explanation of advantages and prognosis from the treating dentist. No effort was made to assign or prescribe treatment because we wanted to evaluate dentistry as it is actually provided by practitioners in a private practice, “real-world” setting. This is a key distinction between efficacy research (outcome observed as a result of ideal treatment) and effectiveness research (outcome observed as a result of treatment as provided in real-world settings).

## Statistical methods

Satisfaction was assessed separately for each of the 14 questions, as well as for the proportion of the 14 questions with which the patient agreed or strongly agreed and whether or not satisfied on all 14 questions. Satisfaction levels were compared across the 10 practitioners, as were proportion of patients who were high-risk and proportion who had a lesion restored. Analyses were performed at the practitioner level using the Wilcoxon rank-sum test to assess statistical significance of findings. As the objective of this phase was to demonstrate the comparability of satisfaction levels for patients receiving non-invasive and invasive treatment, with the number of practitioners already determined, it was designed to be descriptive, not for hypothesis testing. Analyses were conducted using SAS software (SAS v9.4, SAS Institute Inc., Cary NC).

## Results

Each of the 10 practitioners enrolled a maximum of 40 patients (actual range of 17 to 38), for a total of 295 patients enrolled. Restorative data forms were available for 289 patients; 287 patients completed a post-treatment survey – 277 had all 3 forms, but 1 patient survey was missing 13 of 14 questions on satisfaction; analysis is based on 276 patients.

All 10 practitioners were male, general dentists, and owned their dental practice. They had a mean (± SD) years since received dental degree of 23.6 (±6.7), range: 10–32, and mean number of patient visits per week was 42.2 (±22.4), range: 20–90.

Characteristics of the patients, lesions, and treatment are presented in Table 1. The mean (± SD) age was 41.8 (±15.8) years; range 19 to 84 years. Approximately 64% were female, 88% were White, and 79% had at least some dental insurance or third party coverage. Most (71%) patients had moderate caries risk; 54% had only one early caries lesion. A total of 492 lesions were enrolled. The tooth surface that was most commonly affected with early caries was the occlusal surface comprising 70% (*n* = 302) of the early caries lesions in the study. Of the lesions on occlusal surfaces, 14% (*n* = 42, 5 missing depth) were in the inner one-half of the enamel. As shown in Table 1, monitoring and oral hygiene instructions were by far the most commonly recommended treatment, each recommended for 99% of the early caries lesions. Only 57 (13%) lesions were recommended for a restoration (either a full restoration or a preventive resin restoration). Among 244 patients for whom no restoration was recommended, seven patients (total of 13 lesions) chose a restorative treatment anyway. Among these seven patients, three preferred a quick fix to the problem, one did not believe that non-invasive treatment is effective, and four did not provide a reason.

**Table 1:** Characteristics of enrolled patients (*n* = 276), caries lesions (*n* = 429), and treatment

	n	%
Patient characteristics		
Patient’s gender		
Male	99	36
Female	176	64
missing	1	
Patient’s race		
White	242	88
Black/African-American	24	9
Asian	3	1
Other	4	2
missing	3	
Patient’s age group		
19–24 years old	32	12
25–39 years old	103	38
40–54 years old	81	30
55 years old or older	57	21
missing	3	
Patient has any dental insurance		
Yes	218	79
No	58	21
Patient’s caries risk as determined by the practitioner^a^		
Low risk	35	13
Moderate risk	195	71
High risk	46	17
Number of early caries lesions in patient as determined by the practitioner		
1	150	54
2	63	23
3	36	13
4	27	10
Caries lesion characteristics		
Tooth surfaces with early caries		
Occlusal	302	70
Mesial	57	13
Distal	62	15
Buccal or Facial	107	25
Lingual or Palatal	34	8
Incisal	9	2
missing	4	
Practitioner’s preoperative assessment of the deepest part of the early caries lesion		
In the outer one-half of the enamel	345	81
In the inner one-half of the enamel	79	19
missing	5	
Treatment recommended to patient for these lesions^b^		
Monitoring	424	99
Oral hygiene instruction	426	99
Applying or prescribing fluoride	98	23
Applying varnish	9	2
Sealant placement	6	1
Enameloplasty	0	0
Preventive resin restoration	17	4
Full restoration	40	9

^a^Using the American Dental Association Caries Risk Assessment Form (available at the citation for this manuscript at the network’s web site at http://nationaldentalpbrn.org/tyfoon/site/fckeditor/Caries%20Risk%20Assessment%20Form.pdf). A score of 0 indicates that the patient is low-risk. A score of 1–9 means moderate risk. A score of 10 or more indicates high-risk

^b^Multiple choices were possible

As illustrated in Table 2, the percent satisfied on each of the 14 indices ranged from 84 to 99%. Also, Table 3 depicts the mean proportion satisfied was 96%. Overall, 208 (75%) were satisfied with all items; 68 (25%) were dissatisfied in at least one of the 14 satisfaction items, of which 35 (51%) included the aspect of cost, and 2 were not satisfied with any of the 14 items.

**Table 2:** Percent of patients who indicated satisfaction by questionnaire item, overall and separately for each practitioner

	ALL 10 Practitioners			9 Practitioners			Practitioner with low satisfaction scores		
	(*N* = 276 patients)			(*N* = 250 patients)			(*N* = 26 patients)		
Questionnaire item	# satisfied patients (n)	% satisfied	# missing	# satisfied patients (n)	% satisfied	# missing	# satisfied patients (n)	% satisfied	# missing
I am satisfied ….									
.... that I was able to ask questions about my dental treatment	272	99	0	248	99	0	24	92	0
.... that my dentist respects me as a person	271	99	1	247	99	0	24	96	1
.... with the skill of my dentist	268	98	2	247	99	1	21	84	1
.... with how thorough my dentist was	267	97	1	246	99	1	21	81	0
.... with the friendliness of my dentist	266	97	2	244	98	2	22	85	0
.... with the amount of trust that I can place in my dentist	265	96	1	245	98	1	20	77	0
.... was free to make decisions about my dental problems	265	96	0	244	98	0	18	69	0
.... with the treatment I received	260	96	5	238	97	5	22	85	0
.... with how dentist presented options for treatment	264	96	0	245	98	0	19	73	0
.... with the treatment information and handouts provided	264	96	0	244	98	0	20	77	0
.... with the amount of information I received	264	96	0	246	98	0	18	69	0
.... that my dentist understood my concerns	263	95	0	244	98	0	19	73	0
.... with the quality of tooth decay treatment received	262	95	0	245	98	0	17	65	0
.... with the cost of my treatment	241	84	0	218	87	0	23	89	0
Satisfied with ALL	208	75	0	199	80	0	9	35	0

Responses were classified as “satisfied” if patients responded that they “Agree” or “Strongly Agree” with the statement

Denominator is column heading (276, 250, or 26) minus missing

**Table 3:** Summary characteristics by practitioner

		Satisfied on all 14 questions		proportion satisfied		# of high-risk patients		# patients had a lesion restored		# patients had dental insurance	
Dentist	# patients	N	%	mean	SD	N	%	N	%	N	%
A	26	9	35%	80	21	3	12%	4	15%	19	73%
B	17	11	65%	98	4	6	35%	10	59%	14	82%
C	25	17	68%	97	5	4	16%	0	0%	15	60%
D	28	21	75%	98	5	11	39%	7	25%	23	82%
E	38	30	79%	96	16	0	0%	0	0%	24	63%
F	29	23	79%	98	4	1	3%	1	3%	22	76%
G	27	22	81%	95	19	15	56%	1	4%	23	85%
H	37	32	86%	97	12	3	8%	1	3%	37	100%
I	24	21	88%	99	3	7	29%	0	0%	19	79%
J	25	22	88%	99	3	6	24%	8	32%	22	88%
overall	276	208	75%	96	13	46	17%	32	12%	218	76%
All except A	250	199	80%	97	10	43	17%	28	11%	199	80%
A	26	9	35%	80	21	3	12%	4	15%	19	73%
p* =		0.2		0.2		0.9		0.6		0.4	

*Wilcoxon rank sum, 2-tailed for Dentist A compared to all others

One practitioner had notably lower satisfaction scores than the other nine practitioners as shown in Table 2; 35% versus 80% patients were satisfied on all 14 items, and in Table 3, mean proportions satisfied were 80% versus 97%. Lower satisfaction scores for this practitioner were observed on all questions except cost; this score was comparable to that of other practitioners. As evident in Table 3, this practitioner did not appear to have a different patient population in terms of proportion that were high-risk (12% versus 17%) or for whom a lesion was restored (15% versus 11%). The proportion of patients with dental insurance was lower for this practitioner (73% versus 80%).

Neither overall patient satisfaction, nor satisfaction related to costs, differed for patients who received invasive compared to non-invasive treatment; the mean proportion satisfied was 96% for both groups. Adjusting for dental insurance did not affect this, namely, presence of dental insurance was not significantly associated with satisfaction or receiving invasive treatment.

## Discussion

This was a small-scale study in which ten practitioners in one network region recorded patient, lesion, and treatment information about non-cavitated early caries lesions. These practitioners had reported during a questionnaire study about caries diagnosis and caries treatment [26, 38] that they would intervene invasively (i.e., do a dental restoration) on a non-cavitated caries lesion that was only into the enamel. In this study, we quantified patient satisfaction with dental care immediately following therapeutic care for early dental caries. Although results from Phase 4 of this line of research are the focus of this report, findings from the first three phases were also elucidative. Because the only practitioners who participated were those who had previously reported doing invasive treatment for enamel caries, which was subsequently verified during a clinical study, the Phase 1 results were revealing in that these practitioners cited three key barriers to using non-invasive treatment in their practices. These were: (1) patients need help understanding why non-invasive treatment is advantageous; (2) some practitioners fear that if non-invasive treatment leads to an outcome that patients perceive as very negative (e.g., advanced caries), then the patient (or another local dentist from whom the patient decides to begin receiving treatment) will harbor negative attitudes toward the practitioner; (3) practitioners did not want to be seen as outliers, and therefore would like to be able to provide a brochure to help legitimize and validate the non-invasive treatment approach. Additionally, the Phase 1 results guided the design of the informational materials used during the Phase 2 and 3 interventions, making use of the circumstance that these practitioners represented the characteristics of the main target group for the intervention ultimately envisioned after the Phase 4 study had been completed. Another finding was that patient satisfaction levels were the same for patients receiving invasive and those receiving non-invasive therapy. An additional finding was that some patients declined the dentist’s non-invasive treatment suggestion, desiring a surgical treatment approach instead, constituting an informed refusal. While a dentist may believe a surgical approach is not in the patient’s best interest, patient consent is still required prior to the delivery of dental care. However, a dentist knowingly performing invasive treatment when current evidence-based literature supports minimally invasive dentistry raises ethical considerations. The ethical balance between patient autonomy and the practice of evidence-based dentistry can be challenging with these emerging philosophies of care, and the need to consider this balance did not become evident until these patients refused non-invasive treatment during this study. Regardless of treatment type, the lowest satisfaction rating was the item based on patient satisfaction with the cost of treatment (84% satisfaction). The practitioners did not cite costs of non-invasive dental procedures or a lack of dental insurance coverage for non-invasive treatment as barriers to non-invasive therapy in their practices; however, this study found the greatest factor of patient dissatisfaction to be cost. Patient rationale for dissatisfaction with invasive and non-invasive treatment cost is an area worthy of further exploration.

### Factors associated with patient satisfaction

Numerous studies in medicine have focused on patients’ perceptions, and demonstrated that there is a discrepancy between medical professionals’ and patients’ perceptions about the treatment they receive [4, 40, 41]. Patient satisfaction is important because of its link to regular return visits, caregiver trust, perception of technical competence, and treatment outcomes. The dentist-patient relationship has been found to be the most important aspect of patient satisfaction [2]. It is curious that one practitioner in Table 3, practitioner ‘A’, had substantively lower overall satisfaction ratings than the other practitioners. One possible explanation is that he or she felt more uncomfortable in recommending non-invasive therapy, although no measures of this discomfort were included in this study. Several studies have shown that patients evaluate the quality of their dental care based on a range of criteria, particularly their dentist’s interpersonal communication [2, 3, 5, 11, 42–46]. A positive correlation has been found between the dental team’s ability to communicate effectively and patient satisfaction [1–3, 42, 44, 45, 47, 48]. Therefore, a component of the patient satisfaction questionnaire in this study consisted of items enquiring about the patient’s immediate experiences during their dental visit. This includes the perceived interpersonal relationship between patient and dentist that may represent the patient’s view of the skills of the dentist (e.g., a skillful dentist is a painless dentist).

In this study, patient satisfaction was recorded immediately after treatment and patients were not followed longitudinally. About one-fourth of consecutive patients treated for early caries were dissatisfied with some aspect of their dental care experience. Interestingly, mean satisfaction levels of patients who received invasive treatment for early caries did not differ from those who received non-invasive treatment for early caries. This may be due to other factors that lead to patient satisfaction than whether a dentist utilizes a surgical or nonsurgical approach to dental treatment.

All fourteen satisfaction questions in this study received high patient satisfaction ratings. Twelve of the fourteen satisfaction questions received an average satisfaction score of >90%. Two of the fourteen questions received satisfaction endorsement by more than 80% of patients. This study suggests that either the patients were satisfied with the level of technical competence or that their professional needs were addressed satisfactorily; however, the patient’s communication needs regarding cost were met to a lesser degree than technical competence, friendliness and trust.

This study quantified immediate satisfaction with dental care among patients who received treatment for early dental caries and tested the hypotheses that patients who receive non-invasive treatment have higher satisfaction levels than those who receive invasive treatment; and certain patient-level factors are associated with satisfaction. The measure of patient satisfaction was developed specifically for invasive versus non-invasive restorative procedures. Overall, the patient’s satisfaction levels were high. An interesting finding in this study is the similarity in patient satisfaction between invasive and non-invasive dental therapy. This could be explained by the high level of trust a patient places on his/her dentist’s treatment recommendations.

This project has laid important groundwork for designing an intervention to improve early caries treatment by enhancing the translation of research findings into routine clinical practice, making use of the unique advantages of a dental PBRN. The ultimate objective is better oral health at the population level. Achieving patient satisfaction is multifactorial and critical to patient compliance and thus improved oral healthcare. Communication is paramount in achieving patient satisfaction and may hold as much importance to a patient as the technical skill of the dental team. To our knowledge, there is no literature about patients’ declination of non-invasive caries treatment, in favor of invasive treatment.

## Conclusions

About one-fourth of patients treated for non-cavitated early caries were dissatisfied with at least some aspect of their dental care experience. Satisfaction of patients who received invasive treatment did not differ from those who received non-invasive treatment. A small percentage of patients declined the dentist’s recommendation for non-invasive treatment and chose invasive dental treatment instead.

## Appendix

**Table 4:** Patient satisfaction survey questionnaire

	Strongly disagree	Disagree	Neither agree nor disagree	Agree	Strongly agree
1. I am satisfied with how my dentist presented all the options for the treatment of my tooth decay.	1	2	3	4	5
2. I am satisfied with the quality of tooth decay treatment that I received.	1	2	3	4	5
3. I am satisfied with the amount of trust that I can place in my dentist.	1	2	3	4	5
4. I am satisfied with how thorough my dentist was.	1	2	3	4	5
5. I am satisfied with the treatment information and handouts provided.	1	2	3	4	5
6. I am satisfied that my dentist understood my concerns.	1	2	3	4	5
7. I am satisfied with the amount of information about my dental treatment that I received from my dentist.	1	2	3	4	5
8. I feel free to make decisions about my dental problems.	1	2	3	4	5
9. I am satisfied with the skill of my dentist.	1	2	3	4	5
10. I am satisfied that I was able to ask questions about my dental treatment.	1	2	3	4	5
11. I am satisfied with the cost of my treatment.	1	2	3	4	5
12. I am satisfied that my dentist respects me as a person.	1	2	3	4	5
13. I try to take my dentist's advice.	1	2	3	4	5
14. The dentist seemed to know what he was doing during my visit.	1	2	3	4	5
15. I will make changes to my lifestyle if they prevent getting a filling.	1	2	3	4	5
16. The dentist should make the important dental decisions, not me.	1	2	3	4	5
17. I am concerned about feeling pain when I go for dental care.	1	2	3	4	5
18. I am satisfied with the friendliness of my dentist.	1	2	3	4	5
19. Patients should know the cost of their treatment before the treatment begins.	1	2	3	4	5
20. I avoid going to the dentist because I dislike pain.	1	2	3	4	5
21. In general, the fees dentists charge are too high.	1	2	3	4	5
22. I will choose the best treatment for my tooth decay, regardless of the possibility of feeling pain.	1	2	3	4	5
23. Dentists should do more to reduce pain.	1	2	3	4	5
24. Dentists avoid unnecessary patient expenses.	1	2	3	4	5
25. My dentist tried to limit my fear and anxiety.	1	2	3	4	5
26. I will choose the best treatment for my tooth decay regardless of cost.	1	2	3	4	5
27. The dentist respected my opinion about my tooth decay treatment.	1	2	3	4	5
28. I will choose the same treatment for tooth decay on another tooth.	1	2	3	4	5
29. I am satisfied with the treatment I received.	1	2	3	4	5
30. I prefer a filling because it is a quick fix for my tooth decay.	1	2	3	4	5
31. I believe that a treatment to prevent a filling is better than getting a filling.	1	2	3	4	5

## Abbreviations

FDI: Fédération Dentaire Internationale
n: Number
PBRN: Practice-based research network
SD: Standard deviation
